# Targeted Molecular Imaging in Adrenal Disease—An Emerging Role for Metomidate PET-CT

**DOI:** 10.3390/diagnostics6040042

**Published:** 2016-11-18

**Authors:** Iosif A. Mendichovszky, Andrew S. Powlson, Roido Manavaki, Franklin I. Aigbirhio, Heok Cheow, John R. Buscombe, Mark Gurnell, Fiona J. Gilbert

**Affiliations:** 1 Department of Radiology, University of Cambridge and NIHR Cambridge Biomedical Research Centre, Addenbrooke’s Hospital, Cambridge CB2 0QQ, UK; im391@cam.ac.uk (I.A.M.); rm617@cam.ac.uk (R.M.); 2 Metabolic Research Laboratories, Wellcome Trust-MRC Institute of Metabolic Science, University of Cambridge and NIHR Cambridge Biomedical Research Centre, Addenbrooke’s Hospital, Cambridge CB2 0QQ, UK; asp22@medschl.cam.ac.uk; 3 Wolfson Brain Imaging Centre, Department of Clinical Neurosciences, University of Cambridge, Cambridge CB2 1TN, UK; fia20@medschl.cam.ac.uk; 4 Department of Radiology, University of Cambridge School of Clinical Medicine, Addenbrooke’s Hospital, Cambridge CB2 0QQ, UK; heok.cheow@addenbrookes.nhs.uk; 5 Department of Nuclear Medicine, Cambridge University Hospitals, Cambridge CB2 0QQ, UK; john.buscombe@addenbrookes.nhs.uk

**Keywords:** metomidate, nuclear medicine, adrenal, primary aldosteronism, adrenocortical carcinoma

## Abstract

Adrenal lesions present a significant diagnostic burden for both radiologists and endocrinologists, especially with the increasing number of adrenal ‘incidentalomas’ detected on modern computed tomography (CT) or magnetic resonance imaging (MRI). A key objective is the reliable distinction of benign disease from either primary adrenal malignancy (e.g., adrenocortical carcinoma or malignant forms of pheochromocytoma/paraganglioma (PPGL)) or metastases (e.g., bronchial, renal). Benign lesions may still be associated with adverse sequelae through autonomous hormone hypersecretion (e.g., primary aldosteronism, Cushing’s syndrome, phaeochromocytoma). Here, identifying a causative lesion, or lateralising the disease to a single adrenal gland, is key to effective management, as unilateral adrenalectomy may offer the potential for curing conditions that are typically associated with significant excess morbidity and mortality. This review considers the evolving role of positron emission tomography (PET) imaging in addressing the limitations of traditional cross-sectional imaging and adjunctive techniques, such as venous sampling, in the management of adrenal disorders. We review the development of targeted molecular imaging to the adrenocortical enzymes CYP11B1 and CYP11B2 with different radiolabeled metomidate compounds. Particular consideration is given to iodo-metomidate PET tracers for the diagnosis and management of adrenocortical carcinoma, and the increasingly recognized utility of ^11^C-metomidate PET-CT in primary aldosteronism.

## 1. Introduction

The accurate evaluation of adrenal lesions presents a significant challenge for both radiologists and endocrinologists given the high prevalence of incidentally detected nodules on cross-sectional imaging (adrenal incidentalomas (AI)), but also in the context of correctly identifying the culprit lesion in disorders characterized by endocrine hyperfunction (e.g., primary aldosteronism (PA), Cushing’s syndrome (CS), phaeochromocytoma/paraganglioma (PPGL)), as well as in malignant disease, which may be primary adrenal (e.g., adrenocortical carcinoma (ACC), malignant forms of PPGL), or metastatic in origin.

The widespread use and improved performance of cross-sectional imaging techniques, in particular multi-detector computed tomography (MDCT) and magnetic resonance imaging (MRI), for the diagnosis and evaluation of treatment response in many non-adrenal disorders, has increased the chance of detecting previously unsuspected adrenal lesions [1]. The prevalence of AI on CT has been reported between 0.4% and 5% [2,3,4]. Furthermore, their detection is likely to increase with advances in imaging technology, and approach the rates seen in large autopsy series (up to 10% of the population) [5]. Frequently, such nodules are detected on ‘non-adrenal’ imaging protocols, and further investigation (e.g., dedicated adrenal MRI or contrast ‘washout’ CT) is required to determine whether the lesion can be confidently declared as benign, or requires further assessment to exclude/confirm malignancy [1,6,7,8,9,10,11,12,13]. Adrenal adenomas are the most commonly encountered benign lesion and are readily characterized in about 70% of cases by detecting significant amounts of intracellular lipid on unenhanced CT or chemical-shift MRI [1,9]. A further subgroup of adenomas are “lipid-poor” but may be differentiated from other adrenal masses by their rapid washout on contrast-enhanced CT [14,15,16,17]. Other non-malignant adrenal pathologies include myelolipoma, haemorrhage, and simple cysts. Even if a benign adenoma is confidently diagnosed on imaging, screening for endocrine hyperfunction is advised and adrenalectomy may still be necessary to mitigate autonomous hormone secretion [18]. Indeterminate lesions may still be benign, e.g., non-malignant phaeochromocytoma, and confirmed by biochemical testing. Amongst malignant tumours encountered in the adrenal glands, metastases (e.g., from bronchial, renal primary) are the most common, with primary adrenal malignancies (PPGL and ACC) relatively rare; ACC has an estimated incidence of 1–2 cases per million per year [19].

The potential utility of functional imaging for the investigation of adrenal disorders has long been recognized. For example, radionuclide scintigraphy has been used to detect functioning adrenal nodules, but restricted isotope availability and the limited resolution of conventional scintigraphy have proved to be major drawbacks to more widespread use. More recently, ^18^F-fluorodeoxyglucose positron emission tomography-computed tomography (^18^F-FDG PET-CT) has been suggested for the distinction between benign and malignant adrenal disease, but does not distinguish primary adrenal malignancy from metastases [18].

Here, we consider the use of targeted molecular imaging, using tracers (etomidate and in particular its methyl-ester, metomidate) that exhibit specific binding to key enzymes of the adrenal corticosteroid biosynthetic pathway [20]. High levels of expression of some of these enzymes by autonomous adrenal adenomas causing primary aldosteronism (PA), or in ACC, provide a clear rationale for developing tracers based on these molecules for use with PET-CT to allow more accurate localization of the source of hormone excess and/or sites of primary or metastatic ACC.

## 2. Norcholesterol Scintigraphy—Historical Perspective

Traditionally, radiolabeled analogues of cholesterol (^75^Se-selenomethyl-19-norcholesterol (scintadren) and ^131^I-iodomethyl-norcholesterol (NP-59)) have been employed as scintigraphic tracers to identify functionally active adrenal lesions [21,22,23,24]. Although early ^75^Se-selenomethyl-19-norcholesterol studies showed promising results, scintadren is currently no longer available for clinical studies and has been replaced by ^131^I-iodomethyl-norcholesterol (NP-59) [25]. In a declining number of cases, NP-59 continues to be used to investigate the secretory status of adrenal adenomas and aid lesion lateralization [26,27,28,29,30,31]. However, these techniques have significant shortcomings including delivery of a relatively high radiation dose to the adrenal glands, time-consuming acquisition protocols, a requirement for the patient to take a 7–10 day course of high-dose glucocorticoids to suppress non-autonomous hormone synthesis and low sensitivity with poor spatial resolution (traditionally only allowing reliable detection of lesions >2 cm in diameter, although single photon emission computed tomography (SPECT) may afford improved resolution).

## 3. Adrenal Vein Sampling

While a small number of centres continue to use ^131^I-iodomethyl-norcholesterol scintigraphy in the clinical work-up of patients with PA, the technique has largely been replaced by adrenal vein sampling (AVS), in which aldosterone concentrations in effluent blood draining from both adrenal glands are measured together with paired cortisol levels (to generate left and right aldosterone:cortisol (A/C) ratios), which are then compared between sides, with a variety of “thresholds” proposed [32]. However, although considered the gold standard investigation for distinguishing unilateral (aldosterone-producing adenoma (APA)—treated surgically) and bilateral (idiopathic adrenal hyperplasia (IAH)—treated medically) subtypes of PA, this technique also has its challenges: for example, only a small number of centres offer reliable AVS, with many failing to successfully cannulate the right adrenal vein in 25%–50% of cases; in addition, mineralocorticoid receptor antagonists must be withdrawn for up to 6 weeks prior to AVS, placing the patient at risk of uncontrolled hypertension and hypokalaemia. Accordingly, many patients with PA are never considered for surgery because of these limitations in the diagnostic pathway, and reflected in the finding that <300 adrenalectomies are performed per annum in the UK for PA [33,34].

## 4. Development of Metomidate PET for Molecular Adrenal Imaging

The clinical need to more precisely characterize adrenal lesions, coupled with the limitations of traditional scintigraphic techniques and adrenal vein sampling, led to the search for a PET tracer that is concentrated in adrenal tissue, with relatively lower uptake in adjacent organs (e.g., the liver). An important clue to the potential utility of the anaesthetic agent etomidate (and its methyl-ester, metomidate) for this purpose was provided by the observation that clinically relevant hypoadrenalism frequently complicates the use of etomidate in the intensive care setting [35]. While anaesthetic usage of etomidate has fallen significantly since recognition of this adverse effect, it has found a new therapeutic niche as an important adjunct in the management of refractory or severe Cushing’s syndrome, where subhypnotic doses rapidly control hypercortisolism [36].

Etomidate and metomidate are potent inhibitors of the CYP11B1 (11β-hydroxylase) and CYP11B2 (aldosterone synthase) enzymes that are key regulators of adrenal cortisol and aldosterone synthesis, respectively [37]. Given this selectivity for adrenal molecular targets, both were proposed as potential tracers for use in nuclear imaging [38]. In 1998, Bergström and colleagues demonstrated the high affinity of etomidate and metomidate for CYP11B1 and CYP11B2 and their specific binding to the adrenocortical tissue of different species using frozen section autoradiography [20]. The same workers went on to perform preliminary *in vivo* PET imaging in primates and concluded that ^11^C-metomidate was the preferred candidate for clinical imaging due to its shorter synthesis time, higher yield, and specific radioactivity when compared with etomidate [20].

## 5. Synthesis of ^11^C-Metomidate

^11^C-metomidate is synthesised with good radiopharmaceutical yields from the precursor (*R*)-1-(1-phenylethyl)-1H-imidazole-5-carboxylic acid, using ^11^C-methyl iodide for ^11^C labeling [20,39]. The majority of subsequent clinical studies used adaptations of this technique [40,41,42,43]. In 2003, Mitterhauser et al. evaluated three different purification techniques for the routine preparation of ^11^C-metomidate and showed that a particular high performance liquid chromatography (HPLC) purification method resulted in higher purity yields compared to Bergström’s initial approach [44]. Mitterhauser’s method allowed automated preparation of ^11^C-metomidate in activities up to 11 GBq and it enabled an adequate supply of ^11^C-metomidate for two consecutive patients. More recently, we have described the synthesis of ^11^C-metomidate using a captive solvent methylation method and a solution of (*R*)-methyl 1-(1-phenylethyl)-1H-imidazole-5-carboxylic acid in anhydrous dimethylformamide loaded directly into the injector loop of a GE TracerLab FX-C system [45]. This method produces ^11^C-metomidate with a radiochemical purity of greater than 99% and specific activity about 10-fold higher compared to that described by Mitterhauser et al. [45].

## 6. Early Clinical Studies with ^11^C-Metomidate

In the first clinical study to use metomidate, 15 patients with a CT-confirmed unilateral adrenal mass underwent dynamic ^11^C-metomidate PET before surgery or biopsy to correlate findings with subsequent histology [39]. Nine had adrenocortical lesions (six adenomas, of which three were non-functioning; two adrenocortical carcinomas; one nodular hyperplasia) and all showed high tracer uptake. The lesions in the other patients demonstrated virtually no uptake and were subsequently shown not to be adrenocortical in origin (one pheochromocytoma, one myelolipoma, two adrenal cysts, one mesenchymal tumour, and one metastasis). Early images obtained immediately after tracer injection showed high activity in the cortex of the kidney and spleen, with tracer uptake in the adrenocortical lesions increasing throughout the examination. Physiological uptake was high in the normal adrenal glands and stomach, intermediate in the liver, and low in the other abdominal organs.

A subsequent study by Khan et al. focused on the ability of ^11^C-metomidate PET to distinguish adrenocortical tissue in the context of CT-detected recurrence or metastasis in histologically-confirmed ACC [40]. Thirteen studies were performed in eleven patients, of whom six were not taking any medication (steroids or chemotherapy) that could potentially interfere with 11β-hydroxylase activity. The seven others were treated with one or a combination of these drugs. ^11^C-metomidate PET visualized all viable tumours and revealed two additional lesions not identified on CT, while three necrotic adrenocortical cancer lesions were noted to be false negatives by PET and a suspected liver metastasis on CT was a true negative. ^11^C-metomidate PET also correctly excluded a nodal metastasis that was initially suspected on CT. The authors observed large inter-patient variations in tumour SUV_peak_ (5–12) and less uptake of tracer in the tumours of patients treated with corticosteroids and chemotherapy. Nonetheless, the principle that adrenocortical tissue in ACC can be identified by metomidate PET was established.

Two further studies, published in 2004, evaluated the advantages of ^11^C-metomidate PET when compared to ^18^F-FDG PET in the characterization of adrenal incidentalomas [41,46]. Initial biochemical evaluation confirmed the inclusion of a subgroup of patients with functional tumours in both cohorts. In the first one, Minn et al. performed dynamic ^11^C-metomidate PET in 21 patients, followed by static ^18^F-FDG PET imaging in 19 of the 21 [41]. ^11^C-metomidate and ^18^F-FDG uptake was quantified in all lesions and correlated with the hormonal work-up and the size of the lesion on CT. Endocrine evaluation identified seven hormone-secreting adenomas and two phaeochromocytomas, which were confirmed pathologically. Histology defined the remaining lesions as: five non-secretory adenomas, one adrenocortical carcinoma; two benign non-cortical tumors, three malignant non-cortical tumors and one biopsy of normal adrenal tissue. Ranked by ^11^C-metomidate standardized uptake value (SUV), the adrenocortical carcinoma had the highest uptake (SUV = 28.0), followed by functioning adenomas (median SUV = 12.7), non-functioning adenomas (median SUV = 12.2), and non-cortical tumors (median SUV = 5.7). Tumor-to-normal adrenal ^11^C-metomidate SUV ratios were significantly higher in patients with adenomas compared to patients with non-cortical tumors. ^18^F-FDG detected only two out of the three non-cortical malignant lesions, failing to detect adrenal metastases from renal cell carcinoma, while the non-malignant adrenal lesions were either difficult to visualize or not seen at all on ^18^F-FDG PET.

In the second study, Zettinig et al. compared ^11^C-metomidate and ^18^F-FDG PET in 16 patients with adrenal masses (11 adenomas, one adrenal hyperplasia, one adrenocortical cancer, one benign pheochromocytoma, one malignant pheochromocytoma, and one adrenal metastasis from renal cancer) [46]. Nine patients had functioning adrenocortical tumours (Cushing’s syndrome in four, and PA in five subjects, respectively). Two patients had phaeochromocytomas and the remaining five patients had non-functioning adrenal masses. All but one patient underwent surgery. ^11^C-metomidate PET clearly distinguished cortical from non-cortical adrenal masses (median SUVs of 18.6 and 1.9 respectively). Tracer uptake was slightly lower in patients with Cushing’s syndrome compared to patients with PA, but this difference did not reach statistical significance. The single patient with adrenocortical carcinoma had lower ^11^C-metomidate uptake (SUV_max_ = 14.3) compared to the results published by Minn et al., where the highest SUV values were seen in the adrenocortical carcinoma and, furthermore, the highest SUV (32.2) seen in the Zettinig series was in a functioning adenoma. The phaeochromocytomas showed negligible ^11^C-metomidate uptake. In all cases, ^18^F-FDG PET differentiated the malignant from the benign tumors, yielding a sensitivity and specificity of 100%.

In 2006, the Uppsala group followed-up their earlier studies by describing a retrospective series of 73 patients, from whom 75 pathological specimens had been collected, and reported the sensitivity and specificity of ^11^C-metomidate PET to distinguish adrenocortical from non-adrenocortical lesions (twenty-six adenomas, thirteen adrenocortical cancers, eight benign adrenal hyperplasia, six pheochromocytomas, three metastases, and nineteen tumors of non-adrenal origin) [42]. They correlated dynamic ^11^C-metomidate PET findings with histology and lesion size (range 1–20 cm; mean 5.5 cm). ^11^C-metomidate PET correctly identified 42 out of the 47 histologically proven adrenocortical tumours (adenoma or ACC), with 89% sensitivity. In the five false negative ^11^C-metomidate PET studies in subsequently proven adrenocortical tissue, two were of tumours which were found to have extensive necrosis at histology (ACC) and three were thought to be negative as a result of small tumour size (subcentimetre aldosterone producing adenomas (APAs)). Specificity was reported as 96%, with one ‘false-positive’ result in a metastasis erroneously thought to be from an ACC, but which histologically was confirmed as a leiomyosarcoma. Phaeochromocytomas, adrenal metastases, and benign non-adrenocortical lesions were all ^11^C-metomidate PET negative. While the technique could not distinguish benign adrenocortical adenomatous tissue from malignant lesions, this study further established the ability of ^11^C-metomidate PET-CT to distinguish adrenocortical lesions (with a peak SUV 45 min post-tracer injection of >24.3 conferring 95% sensitivity, while a contralateral tumor-to-normal adrenal ratio >1.4 signified a 99.5% chance of an adrenocortical lesion).

In 2009, the same group reported incremental gains in sensitivity and specificity over CT alone for the investigation of AI when MRI was also performed, but with maximum sensitivity and specificity achieved by the further addition of ^11^C-metomidate PET [47]. This study had both prospective and retrospective components and in some cases the diagnosis of an adrenocortical lesion was made after appropriate clinical investigation and follow-up, rather than the gold standard of histology but, despite these potential shortcomings, it adds further evidence to suggest utility for ^11^C-metomidate PET in diagnosing adrenal lesions where standard imaging is equivocal.

## 7. ^123/124/131^I-Iodometomidate Imaging in Adrenal Disease

These early studies in adrenal PET imaging utilized ^11^C-labelled metomidate, which has the disadvantage of a 20-min half-life, requiring the presence of an on-site radiopharmacy and cyclotron for synthesis. They did, however, highlight the validity of molecular imaging targeted to CYP11B1 and B2 to distinguish adrenocortical from non-adrenocortical tissue. Subsequent efforts focused on radiolabeling metomidate with isotopes of iodine, to provide more stable ligands with a longer half-life (permitting longer imaging times and clearance of the tracer to other sites away from the adrenals): ^123^I-iodometomidate for SPECT imaging; ^131^I-iodometomidate as a potential therapeutic radiopharmaceutical agent for treating ACC [48,49,50]; and possibly ^124^I-iodometomidate as a PET tracer (although work with this remains pre-clinical) [48,49,50,51].

In addition, an ^18^F-etomidate analogue, 2-[^18^F]fluoroethyl-desethyl-(*R*)-etomidate (^18^F-FETO), has been evaluated in several *in vitro* and *in vivo* studies [52,53,54]. ^18^F-FETO exhibits high affinity binding to the target enzymes, similar stability to metomidate and etomidate against esterases, and fast *in vivo* metabolism. Several other ^18^F-labelled metomidate analogues have also been proposed as adrenocortical imaging agents, but none have subsequently translated to clinical studies [55]. The only human study of ^18^F-FETO, conducted in ten healthy volunteers, showed very high adrenocortical uptake and physiological distribution in the liver, renal calyces, gallbladder, stomach wall, and pancreas, with only faint uptake in the kidney and bowel [56]. Further clinical studies have, however, been lacking. A new fluorinated PET compound (^18^F-CDP2230), reported to overcome the lack of selectivity of metomidate for CYP11B2 over CYP11B1, was recently described in pre-clinical studies, but has yet to be developed further [57].

Iodo-metomidate tracers have shown more promise. Following the successful radiosynthesis and characterization of ^123/131^I-iodometomidate, Hahner et al. first described the potential of ^123^I-iodometomidate (half-life 13.2 h) for SPECT imaging of adrenocortical lesions expressing CYP11B1 and CYP11B2 [49,58]. In a series of *in vitro*, animal, and human studies, these workers demonstrated ^123^I-iodometomidate’s high binding specificity in tissues of adrenocortical origin, similar pharmacodynamic properties to etomidate/metomidate, lack of toxicity or mutagenicity, and excellent imaging properties in both mice and patients. Four patients (two metastatic adrenal adenocarcinomas, one bilateral adrenocortical adenoma, and one melanoma metastasis) were studied. In each, both normal and adrenocortical tumor tissue were detected within the first 60 min after injection of the radiotracer. The best delineation of the adrenals/lesions was achieved at 4–6 h post-injection and specific uptake was seen exclusively in adrenocortical tissue at 24 h, with very low background activity seen throughout the acquisition window. The adrenal glands, as well as primary and metastatic skeletal lesions of adrenocortical origin, showed high uptake, while a large melanoma metastasis in the left adrenal remained non-avid. The authors highlighted the benefits of ^123^I-iodometomidate SPECT in terms of its short investigation time and low radiation exposure (3–4 mSv), but they also acknowledged the limitations in spatial resolution of ^123^I-iodometomidate SPECT as opposed to ^11^C-metomidate PET for the detection of small adrenal lesions.

In 2013, the same group published the results of a prospective single-center trial on the utility of ^123^I-iodometomidate SPECT for functional characterization of adrenal lesions [59]. 51 patients with adrenal lesions ≥2 cm on CT or MRI underwent sequential planar whole-body ^123^I-iodometomidate imaging (for up to 24 h post-injection) and SPECT/CT (at 4 to 6 h post-injection). Both renal and hepatobiliary tracer uptake were seen, with only transient accumulation in the gallbladder and bowels. The authors used a visual 3-point scoring system to localize tracer activity as either adrenal or extra-adrenal in origin on both SPECT/CT and planar images, resulting in a pooled sensitivity of 89% and a specificity of 85% for differentiating adrenocortical tumors from lesions of non-adrenocortical origin. Semi-quantitative receiver-operating characteristic (ROC) analysis based on ^123^I-iodometomidate retention on planar imaging showed a sensitivity of 83% and a specificity of 86% for identification of adrenocortical lesions at a cut-off SUV tumor-to-liver ratio of 1.3. Although ^123^I-iodometomidate was not able to differentiate between metabolically active benign and malignant primary adrenocortical lesions, its high specificity for identifying adrenocortical tissue, coupled with its suitability for SPECT studies and low radiation dose (2.7 mSv), make it a potential candidate for more widespread clinical application.

Kvaternik et al. recently described the radiosynthesis and biodistribution of ^124^I-iodometomidate as a potential radiopharmaceutical for use in PET imaging [51], thereby avoiding the limitations of SPECT imaging. They reported high production yields with high purity, no radiolysis of the radiopharmaceutical for 48 h at room temperature, and high specific and selective uptake of ^124^I-iodometomidate within the adrenal glands. They concluded that ^124^I-iodometomidate is a promising compound for adrenal PET imaging, but that further studies are needed to explore its clinical utility. It is possible however, that the complex decay patterns of this isotope, may limit its widespread roll out to routine clinical practice.

## 8. ^123/131^I-Iodometomidate Imaging in Adrenocortical Carcinoma (ACC)

Hahner and colleagues have specifically evaluated ^123^I-iodometomidate imaging in 58 patients with histologically-confirmed adrenocortical carcinoma, using the same methodology described previously [50,59]. ^123^I-iodometomidate detected both primary and metastatic ACC. However, tracer uptake was more variable than initially expected. Of the 430 tumours (both adrenal and extra-adrenal metastatic lesions) identified on conventional imaging, 30% showed strong, 8% moderate, and 62% no tracer accumulation. The high percentage of uptake-negative lesions was attributed to a limited ability to detect small lesions (≤2–3 cm), with tumour necrosis or de-differentiation of tumour clones (such that lesions express less CYP11B enzyme—either in progressive tumours or in response to medical treatment), also potentially contributory. Compared to conventional imaging, overall sensitivity and specificity were 38% and 100%, respectively, with 10% of patients harbouring lesions which were ^123^I-iodometomidate avid, but which were not detected on CT. Nonetheless, the fact that around a third of patients with ACC showed specific ^123^I-iodometomidate retention was held not only to confer a diagnostic utility, but also to provide a method of selection of those patients who may respond to ^131^I-iodometomidate targeted radionuclide therapy.

A subset of eleven metastatic ACC patients enrolled in this study also received targeted radionuclide therapy (1.6–20 GBq of ^131^I-iodometomidate in one to three cycles) [48]. One patient showed partial response with 51% reduction in the size of target lesions, five patients had stable disease after treatment, and four patients progressed despite therapy. Median progression-free survival was 14 months (range 5–33 month) with ongoing disease-stabilization at the time of reporting in three patients. Overall, ^131^I-iodometomidate treatment was well tolerated, but several patients showed transient bone marrow suppression, and adrenal insufficiency developed in two patients. The results of this study are notable in showing a long progression-free survival and ongoing disease stabilization, despite a high tumour burden, in patients who had exhausted other treatment options.

## 9. ^11^C-Metomidate PET in Primary Aldosteronism (PA)

PA (a disorder characterized by autonomous aldosterone secretion) is now recognized as an important cause of secondary hypertension (5–10% of all cases), and accounts for 20–25% of refractory hypertension [60,61]. Moreover, for any given blood pressure, it confers additional morbidity and mortality over and above that of “essential” hypertension, reflecting specific adverse effects of aldosterone on the cardiovascular system [62]. Approximately half of all cases of PA are attributable to a unilateral aldosterone-producing adenoma (APA) and, in such cases, adrenalectomy offers the potential for cure of PA and the amelioration/reversal of hypertension [63]. Therefore, a key step in management is the distinction between unilateral and bilateral PA to identify who should be considered for surgery [63]. However, available metrics indicate that currently only a small proportion of potentially eligible patients make it through to adrenalectomy (<300 per annum in the UK) [33]. One major “road block” in the management of PA is the existing ‘gold-standard’ for demonstrating lateralization, which is adrenal vein sampling (AVS). This procedure is invasive and technically challenging, with only a 50–80% success rate for right adrenal vein cannulation, and carries the risk of significant complications [64,65,66]. That AVS is recommended reflects the traditional belief that cross sectional imaging alone is of limited utility in confirming or refuting lateralization: in part this may be due to the presence of a coincidental non-secretory adrenal mass, which although easily visualized, is not the site of aldosterone production [18,63]. A further issue is the small size (<1 cm diameter) of some Conn’s adenomas, which may not be seen using conventional CT and MRI techniques.

### 9.1. Limitations of CT and MRI in PA

Current guidelines reason that thin-slice multi-detector CT and MRI can establish the presence of a unilateral solitary Conn’s adenoma (typically measuring 1–2 cm in diameter) in patients under 35 years who have unequivocal biochemical evidence of PA and an entirely normal contra-lateral adrenal gland, such that these patients can legitimately proceed to surgery without the need for further investigation (reflecting the low prevalence of AI in this subgroup) [63]. For all other patients in whom adrenalectomy is being considered, AVS is viewed as an ‘absolute’ requirement. A further challenge for cross-sectional imaging is presented by the relatively recent recognition that a significant proportion of Conn’s adenomas are subcentimetre in size and may be ”overlooked”, especially when the adrenal gland’s normal contours appear to be preserved (as may occur with microadenomas located within the body of the left adrenal gland). In these circumstances (in which the radiologist reports ”normal adrenals”), many clinicians are reluctant to proceed to further investigation even though lateralizing AVS could be used to support adrenalectomy.

### 9.2. Strategies to Mitigate the Limitations of AVS in PA

The inherent difficulties with AVS have led some workers to seek alternative strategies to establish lateralization in PA including, for example, a Clinical Prediction Score, although crucially, the 100% specificity reported in the original study could not be reproduced by other workers [67,68]. More recently and radically, Dekkers and colleagues, in a randomized trial of CT-versus AVS-driven management, have called into question the added value that AVS confers in terms of patient outcomes (including number of antihypertensive agents required to achieve blood pressure control) following treatment (surgery for a unilateral APA; mineralocorticoid receptor antagonist therapy in bilateral adrenal hyperplasia) [69]. However, importantly, this finding may speak more to the limitations of viewing each investigation in isolation rather than combining assessments of structure and function.

### 9.3. Functional Imaging with ^11^C-Metomidate PET in PA

The need for a more effective, ideally non-invasive, method for diagnosing unilateral PA has therefore become a priority in the face of the rising numbers of patients being recognized with this potentially reversible form of hypertension. The early studies with ^11^C-metomidate PET had established that a return to functional imaging might provide such a solution.

Following the initial demonstrations that APAs are amongst the adrenal tumour subtypes which most avidly concentrate ^11^C-metomidate, several studies have specifically evaluated the utility of ^11^C-metomidate PET in distinguishing PA subtypes [39,41,42,43,45,46,47,70]. Hennings et al. studied eleven patients with biochemically-proven PA and small adrenal adenomas (size range 1–2.5 cm; average 1.7 cm) with dynamic ^11^C-metomidate PET both before and after a 3-day course of oral dexamethasone (mean interval between scans seven days) [43]. The administration of exogenous glucocorticoid is designed to suppress non-autonomous cortisol secretion from the adrenal glands, reducing the expression of CYP11B1 and thus providing greater “contrast” between uptake into normal adrenal tissue and the APA. Two patients with non-functioning adrenocortical adenomas were also included for comparison. ^11^C-metomidate PET reliably identified all adrenal lesions. SUV “hotspot regions of interest” (ROIs, SUV_hs_) were on average higher in the patients with primary aldosteronism than in those with non-functioning lesions. Dexamethasone significantly decreased the SUV_hs_ and SUV_max_ in the normal (contralateral) adrenal, although there was only a trend towards an increase in the tumour-to-normal adrenal ratio within the affected gland, which did not achieve statistical significance. Importantly, the SUV_max_ of the APAs was unaffected by dexamethasone pre-treatment.

In an attempt to improve lesion detection, Razifar and colleagues analyzed pre- and post-dexamethasone ^11^C-metomidate PET studies of seven patients with PA, using masked volume wise principal component analysis (MVW-PCA) [70]. This technique had previously been shown to successfully separate organs and tissues with different kinetic behaviors into their principal components without any prior kinetic assumptions, thus enhancing image quality by reducing noise and increasing contrast between the anatomical structures [71]. In this small study, it was suggested that MVW-PCA provided better definition of regions of interest (ROIs) (i.e., delineation of small APAs) than conventional image summation, in particular when applied to the intermediate pharmacokinetic phase of each dynamic ^11^C-metomidate acquisition. A decrease in tumoural SUV mediated by corticosteroid pre-treatment was also noted to be more evident from time-activity curve (TAC) analysis derived from ROIs drawn on the MVW-PCA images when compared with ROIs defined on the conventionally summed images.

In the largest study published to date, Burton and colleagues evaluated the utility of ^11^C-metomidate PET-CT for establishing lateralization in PA through comparison with the current “gold standard” of AVS in a prospective trial [45,72]. An initial pilot study suggested that pre-treatment for 72-h with dexamethasone (0.5 mg six-hourly for a total of 12 doses) improved the signal to background ratio for detecting APAs. Forty-four patients (39 with PA and five with clinically and biochemically non-functioning AI) underwent a single dynamic PET-CT examination with ^11^C-metomidate. Of the 39 patients with PA, 19 had AVS findings consistent with a unilateral cause for their hyperaldosteronism and a further six were confirmed to have unilateral disease on the basis that they achieved complete biochemical resolution of PA with the cure of their hypertension following unilateral adrenalectomy. The decision to operate in these cases, with inconclusive AVS results, was taken by the patient’s responsible clinician. There were no significant biochemical differences between these patients and those with bilateral PA (by AVS or with bilateral normal or adenomatous adrenal glands; ten patients) at the time of the PET-CT. SUV_max_ was calculated over the final 10 min of data acquisition, commencing 35 min after injection. In patients with unilateral PA (defined by AVS and with a unilateral adenoma), tumour SUV_max_ (21.7 ± 1.6, range 10.3–38.9) was significantly greater than in background normal adrenal tissue (SUV_max_ 13.8 ± 0.6; *p* < 0.00003). Patients with PA and bilateral adrenal hyperplasia/bilateral adenomas had an intermediate SUV_max_ (17.3 ± 1.2), while patients with a non-functioning adenoma had a mean tumor SUV_max_ of 11.5 ± 3.3 (range 0–16.6). An SUV_max_ ratio between tumour and normal adrenal of >1.25 yielded 76% sensitivity and 87% specificity on ROC analysis. Where absolute SUV_max_ was >17, an SUV ratio above 1.25 increased specificity to 100%.

**Figure 1:**
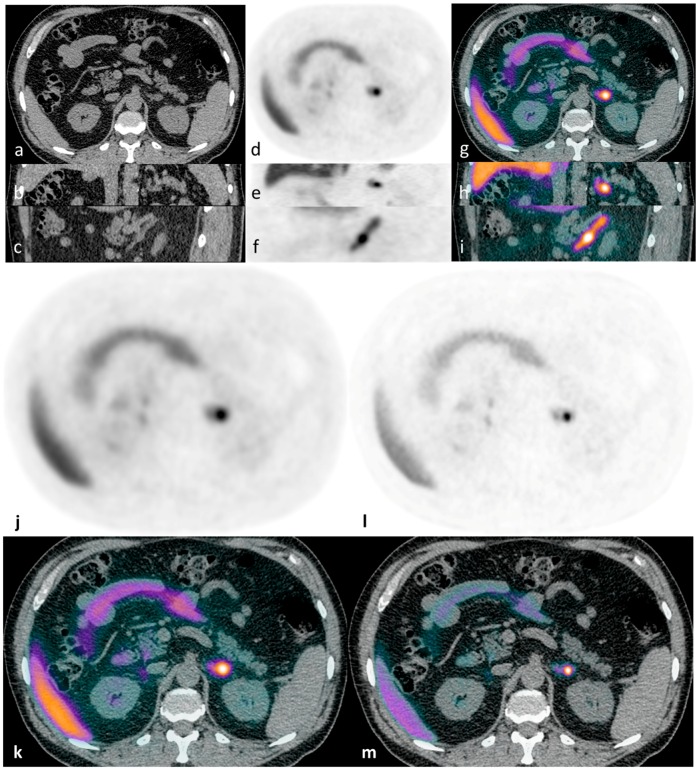
Illustrative case 1: A 65-year-old man with a ten-year history of hypertension and hypokalaemia, requiring multiple agents for blood pressure control (including eplerenone) and oral potassium supplementation, was diagnosed with primary aldosteronism. CT demonstrated a lipid-poor 12 mm left adrenal nodule - displayed in axial, coronal and sagittal planes (**a**–**c**). ^11^C-metomidate PET-CT [(**d**–**f**) (PET) and (**g**–**i**) (PET-CT)] confirmed increased tracer uptake in the left adrenal nodule. The patient underwent laparoscopic left adrenalectomy, with subsequent histology confirming a classical Conn’s adenoma. Post-operatively, he had complete resolution of his hyperaldosteronism with excellent blood pressure control on progressive down-titrating medications (currently two agents with further weaning planned), and with no requirement for supplemental potassium at four-month follow-up. For comparison, axial PET/PET-CT images are shown as reconstructed via “time-of-flight” (TOF) iterative algorithms: without (**j**,**k**) and with resolution modeling (SharpIR) (**l**,**m**).

**Figure 2:**
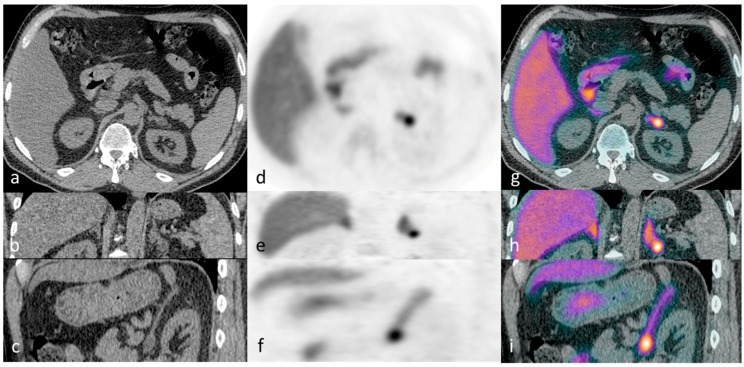
Illustrative case 2: A 55-year-old man with a five-year history of refractory hypertension and hypokalaemia (requiring four agents for blood pressure control) was diagnosed with primary aldosteronism (PA); CT demonstrated a 16mm left adrenal nodule (**a**–**c**), which was ^11^C-metomidate avid, confirming unilateral PA (**d**–**i**). Functional imaging was performed after right adrenal vein cannulation had been unsuccessful during adrenal vein sampling. A left laparoscopic adrenalectomy confirmed a typical Conn’s adenoma. Three years post-operatively his PA remains in complete remission and he requires only a single agent (amlodipine) to achieve full blood pressure control.

**Figure 3:**
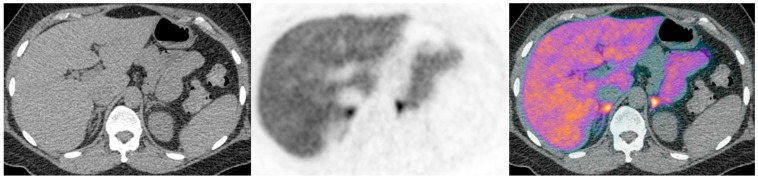
Illustrative case 3: A 42-year-old woman had been diagnosed with hypertension and hypokalaemia 15 years previously in pregnancy. She rotated through trials of various anti-hypertensive medications before achieving reasonable control with spironolactone, together with additional potassium supplementation. A biochemical diagnosis of primary aldosteronism was made following a failure to suppress aldosterone on a saline infusion test. CT findings were equivocal, but with a suggestion of possible subcentimetre nodules bilaterally on CT (**a**). There were no discrete ‘hot nodules’, with symmetrical bilateral uptake on ^11^C-metomidate PET-CT (**b**,**c**). Her bilateral disease is currently managed medically.

**Figure 4:**
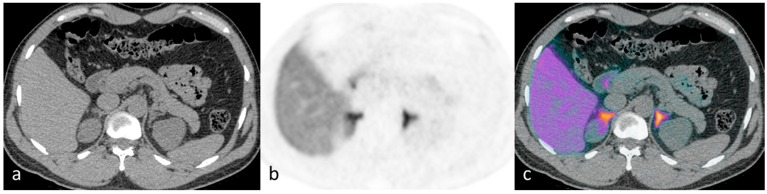
Illustrative case 4: A 40-year-old man with a five-year history of hypertension and hypokalaemia had a diagnosis of hyperaldosteronism confirmed by failure to suppress aldosterone following saline infusion. CT findings were equivocal, showing a possible small right adrenal nodule (**a**). Tracer uptake was symmetrical bilaterally on ^11^C-metomidate PET-CT (**b**,**c**). His bilateral disease is managed medically.

This proof of principle study provided clear evidence that ^11^C-metomidate PET-CT is a valid non-inferior alternative for lateralization in PA. Thereafter, we have gone on to confirm and extend our original findings in a much larger cohort of patients in whom prior AVS has proved technically challenging, inconclusive or not feasible (illustrative cases presented in Figure 1, Figure 2, Figure 3 and Figure 4) (Powlson, Brown, Gurnell, unpublished data). In an important subgroup of these patients with unilateral PA, in whom surgery was not considered appropriate because of the absence of a clear lateralising AVS procedure, ^11^C-metomidate PET-CT has provided unequivocal evidence of unilateral disease and allowed progression to surgery with subsequent demonstration of complete biochemical resolution of PA and a requirement for substantially less, or no, anti-hypertensive therapy (Illustrative cases 1–2). ^11^C-metomidate PET-CT therefore represents an important addition to the investigative algorithm for PA, and seems set to increase the number of patients who will be identified as candidates for potentially curative surgery. Its introduction is timely given the recent recognition that PA is the most common, potentially curable, cause of secondary hypertension.

### 9.4. Optimising ^11^C-Metomidate PET

Current efforts are focused on optimizing data collection and image analysis, and exploring alternative molecular tracers with a longer half-life than ^11^C-metomidate (t ½ = 20 min). The majority of studies conducted with ^11^C-metomidate have included the use of dynamic imaging protocols, with Patlak analysis showing irreversible tracer kinetics consistent with the observed time-activity curves, as well as sustained high uptake approximately 20 min post injection [39,41]. Accordingly, static imaging with an uptake period of at least 20 min combined with SUV analysis is currently recommended for routine clinical use. In our experience, the intravenous administration of 300 MBq of ^11^C-metomidate ensures good tracer uptake within the metabolically active adrenocortical lesion while keeping absorbed patient doses low. The study is performed after a 30-min uptake period and includes low-dose CT for attenuation correction purposes, PET data acquisition, and a diagnostic CT for better lesion localization. The protocol starts with an attenuation correction CT of the upper abdomen (kVp 140; slice thickness 3.75 mm) followed by a 20-min acquisition in a single bed position centered on the adrenal glands on a GE 690 64 slice PET-CT (GE Healthcare, Milwaukee, WN, USA). An unenhanced diagnostic CT to aid lesion localization is performed at the end of the study (kVp 120; slice thickness 1.25 mm). PET images are reconstructed with iterative algorithms using “time-of-flight” (TOF) information without (VPFX^TM^, GE Healthcare, Milwaukee, WN, USA) or with resolution modeling (SharpIR^TM^, GE Healthcare, Milwaukee, WN, USA). Individual data sets are reviewed in axial, coronal, and sagittal planes and then two separate hybrid series are generated by fusing the TOF PET and SHARPIR PET images with the diagnostic CT, respectively.

The major limitation of ^11^C-metomidate is its restricted use to centres with an on-site cyclotron. We and others are therefore currently exploring alternative tracers with a longer half-life, as the next key step towards increasing the availability of functional imaging for patients with PA.

## 10. Conclusions

The development of molecular tracers that can be deployed for the investigation and management of benign and malignant adrenal disease represents a major advance. Adrenocortical carcinoma remains a condition with a dismal prognosis in which existing investigative and therapeutic options are limited. The demonstration that metomidate-labelled compounds can be used both for the detection and potential treatment of metastatic disease opens up new avenues for research in the field of endo-radionuclide therapy. For primary aldosteronism, a condition that is increasingly recognized as accounting for a significant fraction of the adult hypertensive burden, molecular adrenal imaging with metomidate offers a welcome addition to the existing invasive option of adrenal vein sampling, and should allow more patients to progress to the end of the diagnostic pathway, by removing an important road block (“failed AVS”), thus increasing the number of patients who can undergo potentially curative surgery and are spared life-long (anti-hypertensive ± potassium-sparing) medical therapy.
